# A Multicenter, Randomized, Placebo Controlled Trial of HBL Body Lotion for the Treatment of Mild Atopic Dermatitis in China

**DOI:** 10.1111/jocd.70978

**Published:** 2026-06-07

**Authors:** Zhen Duan, Yuning Ding, Liming Wu, Fengming Hu, Zhiqiang Xie, Yuye Li, Fei Hao, Xuewei Liu, Xiguang Liu, Liuqing Chen, Taofeng Liu, Bin Li, Ruiping Wang

**Affiliations:** ^1^ School of Public Health, Shanghai University of Traditional Chinese Medicine Shanghai China; ^2^ Clinical Research Center, Shanghai Skin Diseases Hospital, School of Medicine, Tongji University Shanghai China; ^3^ The First Hospital of Hangzhou Hangzhou China; ^4^ The Skin Diseases Hospital of Jiangxi Nanchang China; ^5^ The Third Hospital Affiliated to Peking University Beijing China; ^6^ The First Hospital Affiliated to Kunming Medical University Kunming China; ^7^ The Third Hospital Affiliated to Chongqing Medical University Chongqing China; ^8^ The First Affiliated Hospital of Henan University of Chinese Medicine Zhengzhou China; ^9^ Heilongjiang Provincial Hospital Harbin China; ^10^ The First Hospital of Wuhan Wuhan China; ^11^ The First Hospital Affiliated to Anhui University of Traditional Chinese Medicine Hefei China

**Keywords:** atopic dermatitis, double blind, emollient, randomized controlled trial

## Abstract

**Background:**

Atopic dermatitis (AD) impairs quality of life due to chronic eczema and pruritus, while conventional emollients as adjunctive therapy show suboptimal treatment efficacy due to inadequate anti‐inflammatory and antipruritic activity.

**Objectives:**

To evaluate the treatment efficacy and safety of HBL incorporated with CAPCS in patients with mild AD.

**Method:**

In this trial, 200 AD patients were randomized (1:1) to receive either the HBL group (HBL incorporated with CAPCS) or the control group (HBL without CAPCS) three times daily for 4 weeks. The primary outcome was the proportion of patients achieving an EASI50 response at week 2. Secondary outcomes included changes from baseline in EASI, DLQI, NRS, and IGA scores at weeks 2 and 4.

**Results:**

At week 2, the HBL group demonstrated significantly higher achievement in EASI score reduction than that in the control group, both for the EASI50 (37.0% vs. 19.0%) and the EASI75 (15.0% vs. 3.0%), and these differences continued into week 4. Greater reductions in EASI, DLQI, NRS, and IGA scores were observed within the HBL group. Adverse events were mild and temporary.

**Conclusion:**

HBL incorporated with CAPCS demonstrates efficacy in improving skin lesions, symptoms, and quality of life in mild AD, supporting its role as an adjunctive therapy.

**Abbreviations**
AD: Atopic dermatitis
AMP: Antimicrobial peptide
CAPCS: Calcium‐based antimicrobial peptide compounds
CRF: Case Report Form
DHEA: Dehydroepiandrosterone
DLQI: Dermatology life quality index
EASI: Eczema Area and Severity Index
FAS: Full analysis set
HBL: Haidebao body lotion
IQR: Interquartile range
NRS: Numeric rating scale
PPS: Perprotocol set
SD: Standard deviation

## 1 Background

Atopic dermatitis (AD) is a prevalent chronic inflammatory skin disease across a broad age range, imposing a substantial burden on patients and caregivers. This burden stems from its unpredictable course, visible lesions, intense pruritus, sleep disruption, and associated comorbidities, all of which severely impair quality of life and psychosocial well‐being [1, 2]. Converging evidence points to a multifactorial pathogenesis, centered on the triad of epidermal barrier impairment, immune dysregulation, and microbial dysbiosis [3]. Although mild AD often presents with localized symptoms such as erythema, xerosis, and pruritus, inadequate long‐term management can precipitate progression to more severe disease forms and heighten the risk of the “atopic march,” thereby predisposing patients to comorbid conditions like allergic rhinitis and asthma [4]. Current treatments for mild AD include corticosteroids, calcineurin inhibitors, and emollients. However, Long‐term use of corticosteroids is associated with adverse effects such as skin atrophy and telangiectasia, while calcineurin inhibitors can include local reactions like burning and itching [5, 6]. These limitations underscore a significant unmet need for safer, targeted therapies in mild AD, a subgroup representing approximately 60% of patients [7].

Emollients serve as a cornerstone of AD management by restoring barrier integrity, reducing transepidermal water loss, and relieving pruritus [8, 9]. Genetic and environmental factors exacerbate stratum corneum protease activity, damaging the skin barrier in AD; consequently, as a barrier repair therapy, emollients serve as the treatment mainstay by prolonging relapse intervals, attenuating acute flares with a steroid‐sparing effect, and potentially preventing AD onset when used from birth [6]. The development of novel active ingredients has further enhanced their reparative, anti‐inflammatory, and moisturizing benefits, especially for mild AD [10, 11].

The calcium‐based antimicrobial peptide compounds (CAPCS) are a novel topical formulation composed of naturally derived active calcium and antimicrobial peptides (AMPs) extracted from farmed marine shellfish. The epidermal calcium gradient is essential for regulating keratinocyte differentiation, barrier formation, and cutaneous homeostasis [12]. The AMP component in CAPCS increases bacterial membrane permeability, enabling rapid calcium influx. This elevated intracellular calcium disrupts cellular metabolism, causes organelle leakage, and ultimately leads to bacterial death [13]. Although preliminary evidence suggests that Haidebao Body Lotion (HBL) incorporating CAPCS offers clinical benefits for mild AD, robust evidence from high‐quality trials is lacking. To address this gap, we designed this multicenter, double‐blind, randomized, placebo‐controlled study to rigorously evaluate the efficacy and safety of HBL incorporated with CAPCS as an adjunctive therapy for mild AD.

## 2 Methods

### 2.1 Study Design

This multicenter, double‐blind, randomized, placebo‐controlled trial was conducted across 10 tertiary hospitals in China from October 2023 to June 2025, adhering to the approved protocol [14]. The study was approved by the institutional ethics committee and registered in a public database. Informed consent was obtained from all individual participants included in the study.

Participants with mild AD (*N* = 200) were randomized (1:1) to either the HBL group (containing CAPCS) or the control group (HBL without CAPCS) using block randomization with block sizes of 4 and 6 to ensure allocation concealment. Both participants and investigators were blinded to the treatment assignment. The allocated intervention was administered three times daily for 4 weeks. To maintain blinding, the application procedure and individual guidance were identical between groups. Any patient requiring emergency additional treatment was withdrawn from the study, with the event fully documented. At baseline (day 0), all participants underwent physical examination and skin lesion assessment, followed by efficacy evaluations at weeks 2 and 4. Further methodological details are available in the published protocol [14].

### 2.2 Participants

Participants aged 18–55 years with physician‐diagnosed AD by Williams criteria, a single target lesion 2–10 cm on the trunk or extremities, baseline EASI below 6 and less than 5% BSA involvement were enrolled. Exclusion criteria included pregnancy or lactation, systemic treatment in the preceding 3 months or topical/phototherapy in the prior 2 weeks, serious systemic diseases, calcium supplement allergy, systemic diseases or active skin conditions affecting evaluation, scars, birthmarks, tattoos, freckles, bacterial/viral/fungal infections, participation in other clinical trials during the previous month, and communication impairments that would compromise questionnaire completion.

### 2.3 Outcomes

The primary endpoint was the percentage of participants achieving EASI50 response at week 2, with EASI50 defined as ≥ 50% improvement in EASI score from baseline and calculated as (EASI at baseline—EASI at week *t*)/(EASI at baseline) × 100%. Secondary endpoints comprised absolute changes in EASI [15], NRS [16], IGA [17], DLQI [18] scores from baseline to weeks 2 and 4. Safety assessments were conducted throughout the study period, including adverse events, and laboratory abnormalities. Any adverse events were accurately recorded and treated by dermatologists.

### 2.4 Sample Size and Statistical Analysis

In accordance with our pre‐specified sample size calculation [14], a target of 200 patients with mild AD was set for this study. Efficacy and safety analyses followed the intention‐to‐treat principle, comprising every participant who was randomly assigned. Missing primary outcome data were assumed to be missing at random and were imputed by carrying the last observation forward. All analyses were performed with SAS 9.4, using a two‐tailed alpha level of 0.05 for statistical significance.

In this study, normality of continuous variables was assessed with the Shapiro–Wilk test, with a normal distribution defined as *p* > 0.05. Normally distributed data are summarized as mean ± standard deviation (SD), while skewed data are reported as median (interquartile range, IQR). Categorical variables are expressed as frequency (*n*) and percentage (%). Continuous variables were compared using the Student's *t*‐test (normal distribution) or the Mann–Whitney *U* test (non‐normal distribution), depending on their distribution, and reported with *p* values and applicable 95% confidence intervals. Categorical variables were compared using the chi‐square test, with statistical significance set at a two‐tailed *p* < 0.05.

## 3 Results

### 3.1 Patient Characteristics

**FIGURE 1:**
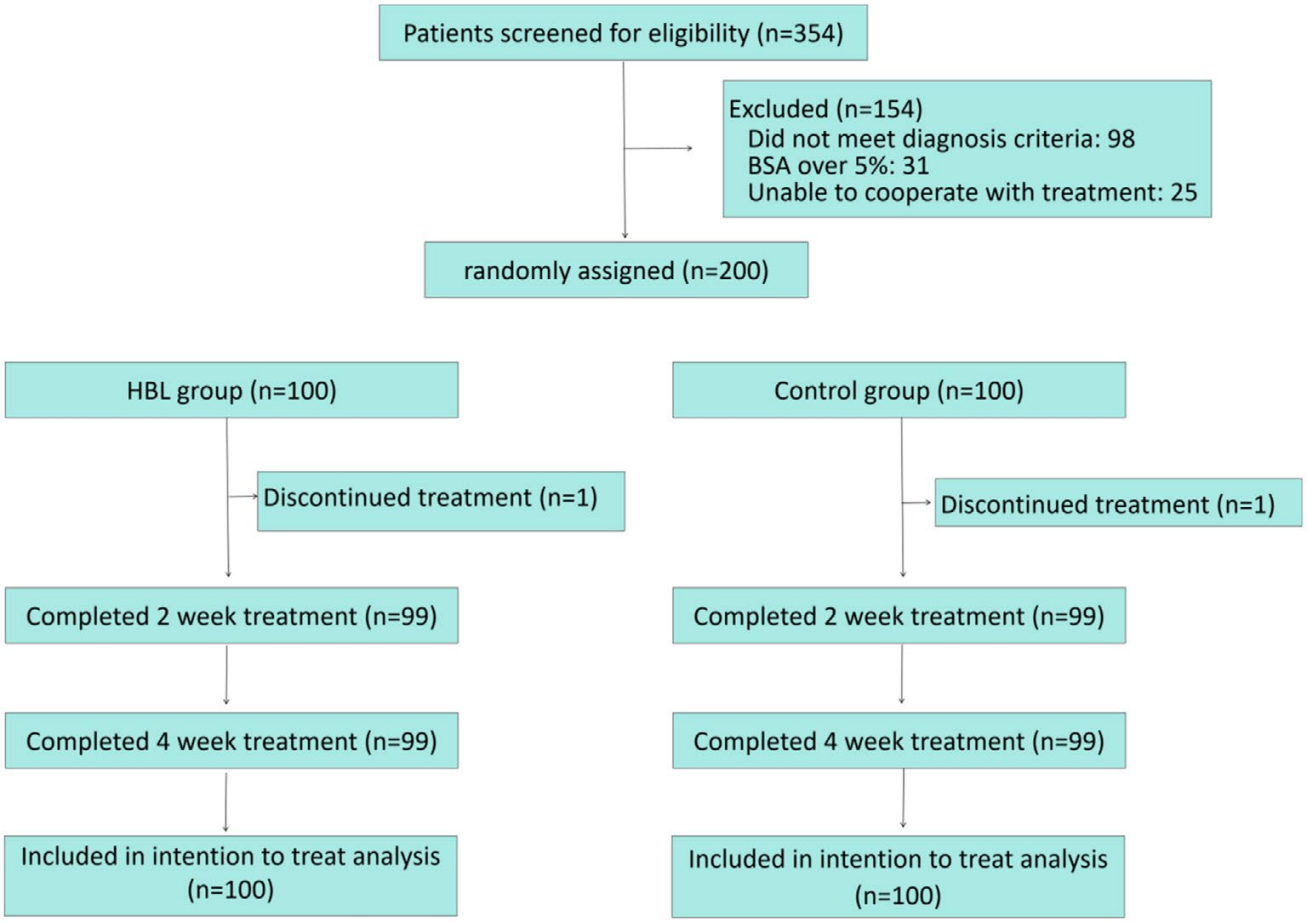
The flow diagram of the clinical trial.

Between October 2023 and June 2025, there were 354 patients screened in 10 study sites. After 154 patients were excluded, the ITT analysis included 200 patients with mild AD (100 in the HBL group and 100 in the control group). A total of 2 (1.0%) patients dropped out during this study, including 1 (1.0%) in the HBL group and 1 (1.0%) in the control group (Figure 1). The baseline features of mild AD patients are shown in Table 1. There were no significant differences between the HBL group and the control group in any demographic characteristics, disease severity, or AD lesion location, only except for patients with AD location involving the face and head. The proportion of AD location in “face and head” was 30.0% (*n* = 30) in the HBL group, which was higher than that in the control group (*n* = 17, 17.0%).

**TABLE 1:** The baseline characteristics of patients with atopic dermatitis (AD).^a^

Characteristics	HBL (*n* = 100)	Control (*n* = 100)	*p* value
Age (years), mean (SD)	36.0 (11.2)	37.1 (11.9)	0.496
Age group (*n*, %)			0.176
< 35 years	44 (44.0)	48 (48.0)	
35–45 years	30 (30.0)	19 (19.0)	
46–60 years	26 (26.0)	33 (33.0)	
Gender (*n*, %)			0.087
Male	49 (49.0)	37 (37.0)	
Female	51 (51.0)	63 (63.0)	
Residency status (*n*, %)			1.000
Urban	72 (72.0)	72 (72.0)	
Rural	28 (28.0)	28 (28.0)	
Marital status (*n*, %)			0.989
Married	39 (39.0)	38 (38.0)	
Unmarried	60 (60.0)	61 (61.0)	
Divorced/widow/widower/others	1 (1.0)	1 (1.0)	
BMI (kg/m^2^)^b^, mean (SD)	23.4 (3.6)	22.8 (4.0)	0.222
BMI (kg/m^2^)^b^, *n* (%)			0.518
< 23.9 (low or normal weight)	7 (7.0)	8 (8.0)	
24.0–28.0 (overweight)	52 (52.0)	61 (61.0)	
> 28.0 (obesity)	41 (41.0)	31 (31.0)	
AD location, *n* (%)			
Face and head	30 (30.0)	17 (17.0)	0.030
Upper limb	51 (51.0)	47 (47.0)	0.572
Lower limb	44 (44.0)	51 (51.0)	0.322
Body trunk	37 (37.0)	40 (40.0)	0.663
EASI at week 0, median (IQR)^c^	1.6 (1.0–2.8)	1.6 (1.2–2.2)	0.324
DLQI at week 0 (%), median (IQR)^d^	8.0 (4.0–12.5)	7.0 (3.0–11.0)	0.356
IGA at week 0, median (IQR)^e^	2.0 (2.0–2.0)	2.0 (2.0–2.0)	0.202
Level of Pruritus at week 0, median (IQR)	4.0 (2.0–5.0)	4.0 (3.0–5.0)	0.988

Abbreviations: BMI, body mass index; DLQI, dermatological life quality index; EASI, eczema area and severity index; IGA, Investigator's Global Assessment; IQR, Interquartile range; SD, standard deviation.

^a^ Data represented as mean (SD), median (IQR) or frequency (%) as appropriate.

^b^ BMI was calculated as weight in kilograms divided by height in meters squared.

^c^ EASI indicates the severity of AD, with a higher value representing a more severe condition (0 [better]–72 [worse]).

^d^ DLQI score indicate the life quality affected by skin diseases among patients, with a higher value represents a more severe condition (0 [better]–30 [worse]).

^e^ IGA indicate the investigator's overall evaluation of AD, with a higher score representing a more severe condition (0 [better]–5 [worse]).

### 3.2 Primary Outcome

In Table 2, the EASI50 response rate was 37.0% in the HBL group and 19.0% in the control group; the difference was statistically significant. The mean EASI score was 1.3 (SD = 1.0) at week 2 in the HBL group and 1.6 (SD = 1.3) in the control group (between‐group difference, −0.4 [95% CI, −0.7 to −0.1]). The change of EASI score from baseline to week 2 in the HBL group was 0.8 points (SD = 1.0) and 0.2 points (SD = 1.2) in the control group (between‐group difference, 0.6 [95% CI, 0.2 to 0.8]). At week 2, the HBL group demonstrated significantly higher response rates than the control group, with differences between the HBL group and the control group in EASI60 (25.0% vs. 5.0%) and EASI75 (15.0% vs. 3.0%) all being statistically significant.

**TABLE 2:** Primary and secondary outcomes of patients with treatment in HBL and control group.^a^

Variables	HBL (*n* = 100)	Control (*n* = 100)	Difference mean (95% CI)	*p* value
Primary outcome				
EASI at week 2, mean (SD)	1.3 (1.0)	1.6 (1.3)	−0.4 (−0.7 to −0.1)	0.026
EASI decreased at week 2, median (IQR)	0.5 (0.1 to 1.2)	0.4 (0.0 to 0.8)	NA	0.005
EASI decreased at week 2, mean (SD)	0.8 (1.0)	0.2 (1.2)	0.6 (0.2 to 0.8)	0.001
EASI 50 at week 2, *n* (%)^b^	37 (37.0)	19 (19.0)	NA	0.005
EASI 60 at week 2, *n* (%)^c^	25 (25.0)	5 (5.0)	NA	0.001
EASI 75 at week 2, *n* (%)^d^	15 (15.0)	3 (3.0)	NA	0.003
Secondary outcomes				
EASI at week 4^e^				
EASI score, mean (SD)	0.9 (1.0)	1.5 (1.7)	−0.6 (−1.0 to −0.2)	0.003
EASI score, median (IQR)	0.6 (0.2 to 1.2)	0.9 (0.5 to 1.6)	NA	0.005
EASI score decreased, mean (SD)	1.1 (1.5)	0.3 (1.7)	0.8 (0.3 to 1.2)	0.001
EASI decreased, median (IQR)	0.9 (0.4 to 1.8)	0.6 (0.0 to 1.2)	NA	0.001
EASI 50, *n* (%)	65 (65.0)	41 (41.0)	NA	0.001
EASI 75, *n* (%)	42 (42.0)	19 (19.0)	NA	0.001
IGA score, mean (SD)^f^				
IGA score at week 2	1.4 (0.5)	2.1 (0.8)	−0.7 (−0.9 to −0.5)	0.001
IGA score at week 4	1.1 (0.7)	1.5 (0.7)	−0.4 (−0.6 to −0.2)	0.001
IGA score decreased at week 2	0.8 (0.8)	0.0 (0.9)	0.8 (0.5 to 1.0)	0.001
IGA score decreased at week 4	1.1 (0.9)	0.6 (0.9)	0.5 (0.2 to 0.8)	0.001
NRS score, mean (SD)^g^				
NRS score at week 2	1.9 (1.1)	2.9 (1.3)	−0.9 (−1.3 to −0.6)	0.001
NRS score at week 4	1.0 (0.8)	2.3 (1.1)	−1.3 (−1.6 to −1.1)	0.001
NRS score decreased at week 2	1.9 (1.9)	1.1 (2.0)	0.9 (0.4 to 1.4)	0.001
NRS score decreased at week 4	2.9 (2.0)	1.6 (2.0)	1.3 (0.7 to 1.8)	0.001
DLQI score, mean (SD)^h^				
DLQI score at week 2	3.7 (2.7)	6.1 (5.0)	−2.4 (−3.5 to −1.3)	0.001
DLQI score at week 4	2.1 (2.1)	4.6 (4.8)	−2.5 (−3.5 to −1.5)	0.001
DLQI score decreased at week 2	5.1 (5.2)	2.3 (4.0)	2.9 (1.6 to 4.2)	0.001
DLQI score decreased at week 4	6.7 (5.0)	3.7 (5.5)	3.0 (1.5 to 4.5)	0.001

Abbreviations: BMI, body mass index; DLQI, dermatological life quality index; EASI, eczema area and severity index; IGA, Investigator's Global Assessment; IQR, Interquartile range; SD, standard deviation.

^a^ Data represented as mean (SD), median (IQR) or frequency (%) as appropriate.

^b^ EASI_50_ is defined as patients achieving at least 50% improvement in EASI score and calculated by the formula [(EASI at baseline—EASI at week *t*)/EASI at baseline] × 100%.

^c^ EASI_60_ is defined as patients achieving at least 60% improvement in EASI score and calculated by the formula [(EASI at baseline—EASI at week *t*)/EASI at baseline] × 100%.

^d^ EASI_75_ is defined as patients achieving at least 75% improvement in EASI score and calculated by the formula [(EASI at baseline—EASI at week *t*)/EASI at baseline] × 100%.

^e^ EASI indicate the severity of AD, with a higher value represents a more severe condition (0 [better]–72 [worse]).

^f^ IGA indicates the investigator's overall evaluation of AD, with a higher score representing a more severe condition (0 [better]–5 [worse]).

^g^ NRS indicates the pruritus level of atopic dermatitis patients, with a higher score representing a more severe condition (0 [better]–10 [worse]).

^h^ DLQI score indicate the life quality affected by skin diseases among patients, with a higher value representing a more severe condition (0 [better]–30 [worse]).

### 3.3 Secondary Outcomes

Similar results of EASI score improvement were observed at week 4, the change of EASI score was greater in the HBL group than in the control group (Table 2). HBL group achieved higher EASI50 and EASI75 response rates than that in the control group (65.0% vs. 41.0% for EASI50 and 42.0% vs. 19.0% for EASI75, respectively) at week 4. HBL group reduced more in IGA score at week 2 and week 4 than that in the control group (between‐group difference, 0.8 [95% CI: 0.5 to 1.0] at week 2; 0.5 [95% CI: 0.2 to 0.8] at week 4). Change of NRS scores from baseline was greater in HBL group than in the control group (between‐group difference, 0.9 [95% CI: 0.4 to 1.4] at week 2; 1.3 [95% CI: 0.7 to 1.8] at week 4). Compared with the control group, HBL group showed significantly greater decreases in DLQI scores (between‐group difference, 2.9 [95% CI: 1.6 to 4.2] at week 2; 3.0 [95% CI: 1.5 to 4.5] at week 4, Table 2).

### 3.4 Sub‐Group Analysis

**FIGURE 2:**
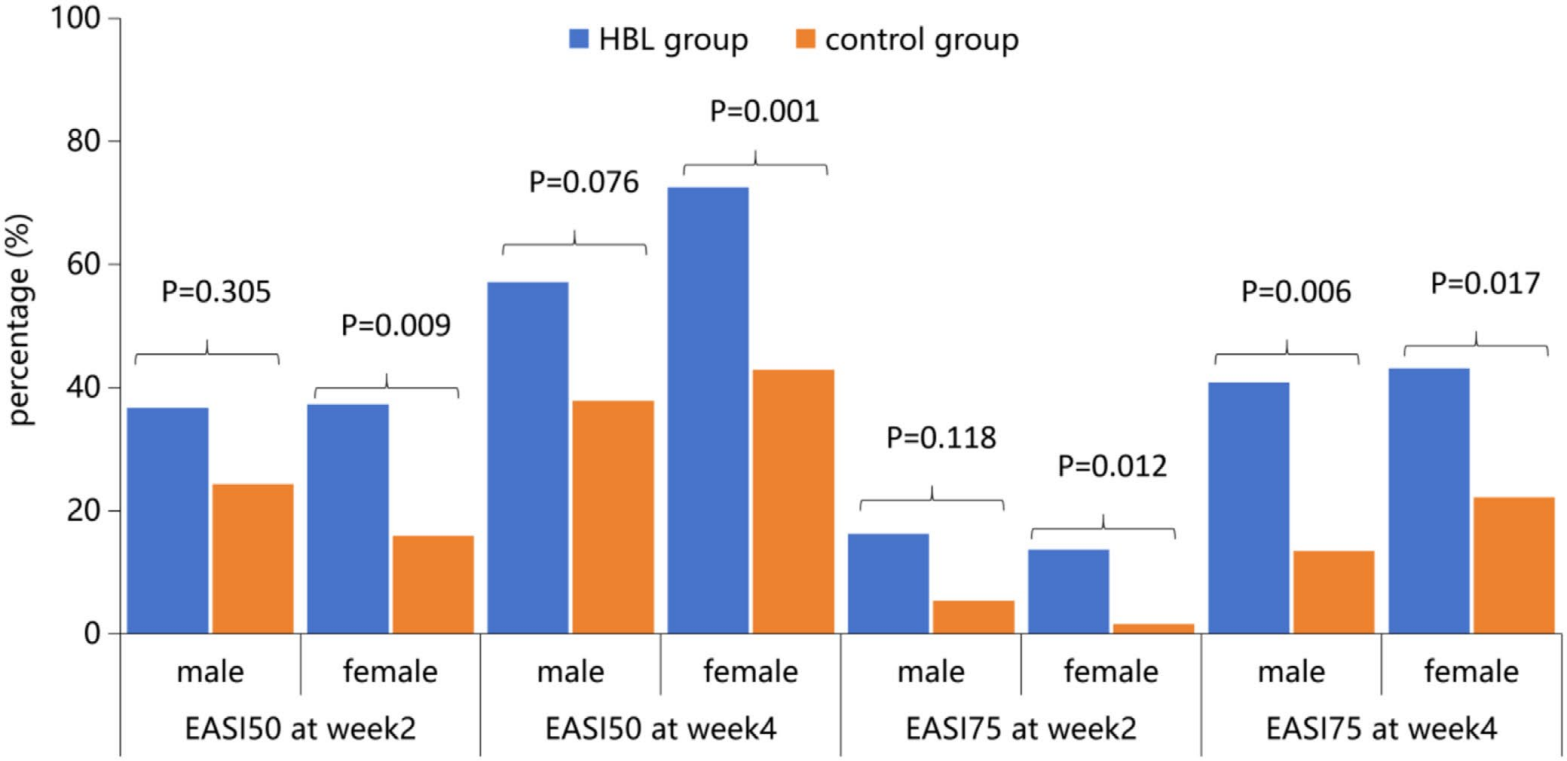
EASI50 and EASI75 achievement rates by sex subgroups in HBL and control groups.

In this study, we performed subgroup analyses to compare the EASI50 and EASI75 response rates between the HBL group and control group by sex at week 2 and week 4. Data in Figure 2 indicated that both male and female patients with mild AD in the HBL group had greater EASI50 and EASI75 response rates than in the control group, and were statistically significant in EASI50 and EASI75 both at week 2 and week 4 among female patients, and EASI75 at week 4 among male patients.

### 3.5 Adverse Event

As shown in Table 3. Adverse effects occurred in 1.0% (*n* = 1) of patients in the HBL group and 1.0% (*n* = 1) in the control group, without between‐group difference (*p* = 1.000). No patients withdrew from the study due to adverse effects and no serious adverse event occurred in the trial.

**TABLE 3:** Adverse events among patients with AD in HBL group and control group.^a^

Variables	HBL (*n* = 100)	Control (*n* = 100)	*p* value
Adverse events, *n* (%)			1.000
Yes	1 (1.0)^a^	1 (1.0)^b^	
No	99 (99.0)	99 (99.0)	
Severe adverse events, *n* (%)			1.000
Yes	0 (0.0)	0 (0.0)	
No	100 (100.0)	100 (100.0)	

Abbreviations: AD, atopic dermatitis; HBL, HaiDeBao body lotion.

^a^ A 40‐year‐old married female, weighing 65 kg and standing at 170 cm, visited on January 11, 2025. Her baseline scores included an EASI of 3.8, DLQI of 13, and IGA of 3. Following a cold on January 20, her symptoms deteriorated, yet treatment was not discontinued by the physician. After 2 weeks of treatment, her scores were revised to an EASI of 5.8, DLQI of 15, and IGA of 3AD. By the fourth week, the EASI score had decreased to 3 points, DLQI to 6 points, and IGA to 3 points; however, clinical judgment deemed the treatment ineffective.

^b^ A 19‐year‐old unmarried male, weighing 80 kg and standing at 180 cm, was admitted on June 28, 2024. His baseline scores were an EASI of 1.2, DLQI of 3, and IGA of 2. After 2 weeks of treatment, his scores slightly changed to an EASI of 1.5, DLQI of 2, and IGA remaining at 2, indicating no significant improvement. Continued treatment led to scores of an EASI of 2.7, DLQI of 6, and IGA of 3 after 4 weeks. On July 20, clinical judgment revealed worsening symptoms, with skin lesions exhibiting erosion, exudation, and facial erythema. The treatment was deemed ineffective, prompting subsequent administration of Dupilumab treatment.

## 4 Discussion

This first nationwide, multi‐center, randomized, placebo‐controlled study evaluates the efficacy and safety of HBL incorporated with CAPCS for mild AD in a Chinese population. During a 4‐week treatment period, patients received HBL incorporated with CAPCS, which was associated with a significantly higher EASI50 response rate than that in the control group at week 2, and the effects continued at week 4 following the treatment. The incidence of adverse events was low, without patients withdrawing from the study due to HBL related adverse reactions. Our findings align with established research on the therapeutic efficacy of CAPCS for AD.

In this study, patients in the HBL group and control group were comparable at baseline, except for the fact that the HBL group had a higher proportion of patients with facial and head AD lesions. Findings in this study indicated that patients in the HBL group achieved greater and sustained benefits both in the primary and the secondary outcomes. Prolonged use of moderate‐to‐high‐potency topical corticosteroids on sensitive skin like the face is associated with adverse effects including perioral dermatitis, telangiectasia, acne, and erythema. Moreover, long‐term application of potent topical corticosteroids to the eyelids is associated with cataracts and glaucoma [19]. In recent years, studies have identified the face and head as common sites of AD involvement, with lesions in these areas having a significant impact on patients' quality of life. So, there is a need for additional effective and well‐tolerated topical therapies to treat AD in the face and neck [20, 21].

The EASI score is a standard scale for assessing the severity of AD. In this study, the change of EASI score reduction from baseline was significant both at week 2 and week 4. Moreover, the HBL group had significantly higher response rates of EASI50, EASI60, and EASI75 than that in the control group, which further confirmed the clinical superiority of HBL treatment. Similar to the EASI score, the reduction in DLQI, NRS, and IGA in the HBL group was also greater than that in the control group, both at week 2 and week 4. These results suggested that HBL incorporated with CAPCS could not only reduce the skin lesions and pruritus intensity, but also improve the quality of life in patients with mild AD. Pruritus, often accompanied by erythematous and scaly lesions, is the most common symptom of AD patients [22]. In this study, the significant reduction in NRS score after treatment indicated that HBL incorporated with CAPCS could alleviate pruritus among AD patients, which was crucial for AD treatment due to the interruption of the itch‐scratch cycle could mitigate AD signs, enhance quality of life, and prevent AD progression [23].

In this study, subgroup analysis indicated that female patients with mild AD achieved a more significantly higher response rate of EASI50 and EASI75 at week 2 and week 4 than male AD patients; this might be due to the gender disparity in hormone. Estrogen in female patients enhances the activity of Th2 cells and regulatory T cells, up‐regulates the expression of endogenous AMPs such as LL‐37, and directly promotes skin barrier repair [24, 25]. The AMP components in CAPCS can synergize with endogenous AMPs, while the calcium ions modulate epidermal calcium gradients, creating mutual reinforcing effects with estrogen‐mediated barrier repair. The higher level of DHEA in females may also further enhance the therapeutic efficacy by modulating Th2 responses, whereas the broad immune‐suppressive effects of androgens may attenuate treatment responses in male patients [26, 27].

The primary strength of this trial lies in its rigorously executed multicenter, double‐blind, randomized, and placebo‐controlled design across 10 cities in mainland China. The use of a meticulously matched placebo ensured the integrity of blinding, yielding high‐quality evidence for the efficacy of HBL combined with CAPCS. Importantly, our results establish this combination as a viable and effective non‐hormonal alternative, addressing a crucial need for safe long‐term management strategies in mild AD.

Several limitations of this study warrant consideration. Firstly, the 4‐week intervention period precludes assessment of the long‐term efficacy and relapse‐prevention potential of HBL with CAPCS. Secondly, despite the multicenter design enhancing generalizability, unmeasured confounding from regional variations in climate, lifestyle, and environmental exposures cannot be entirely ruled out. Future research should proactively collect and adjust for these factors. Finally, the therapeutic mechanisms underpinning the observed clinical benefits remain unelucidated. The lack of mechanistic insight confines our interpretation of response heterogeneity, notably the gender differences identified in subgroup analyses.

## 5 Conclusion

HBL incorporated with CAPCS demonstrates efficacy in improving skin lesions, symptoms, and quality of life in mild AD, supporting its role as an adjunctive therapy.

## Author Contributions

Zhen Duan, Yuning Ding, and Ruiping Wang developed the draft of the manuscript. Ruiping Wang revised the manuscript. Zhen Duan, Yuning Ding, Ruiping Wang, Liming Wu, Fengming Hu, Zhiqiang Xie, Yuye Li, Fei Hao, Xuewei Liu, Xiguang Liu, Liuqing Chen and Bin Li contributed to writing all sections of the manuscript and critically reviewed and approved the final version.

## Funding

This study was supported by China Haidebao program (LCIIT202314), the Clinical Research Plan of SHDC (SHDC2022CRS053, SHDC2024CRX032), and the Clinical Research Program of Shanghai Municipal Health Commission (202240371). These funders had no role in the study design, data collection and analysis, decision to publish, or manuscript preparation.

## Ethics Statement

This randomized controlled study was approved by the Ethics Committee of Shanghai Dermatology Hospital, and the study methods were conducted in accordance with the approved standards.

## Consent

All the patients have been informed and signed informed consent before the study.

## Conflicts of Interest

The authors declare no conflicts of interest.

## Data Availability

Research data are not shared.
